# Altered chromatin organization and SUN2 localization in mandibuloacral dysplasia are rescued by drug treatment

**DOI:** 10.1007/s00418-012-0977-5

**Published:** 2012-06-17

**Authors:** Daria Camozzi, Maria Rosaria D’Apice, Elisa Schena, Vittoria Cenni, Marta Columbaro, Cristina Capanni, Nadir M. Maraldi, Stefano Squarzoni, Michela Ortolani, Giuseppe Novelli, Giovanna Lattanzi

**Affiliations:** 1 Institute of Molecular Genetics, Unit of Bologna IOR, National Research Council of Italy - CNR, Via di Barbiano 1/10, 40136 Bologna, Italy; 2 Laboratory of Musculoskeletal Cell Biology, RAMSES, Rizzoli Orthopedic Institute, Via di Barbiano 1/10, 40136 Bologna, Italy; 3 Department of Biopathology and Diagnostic Imaging, University of Tor Vergata, Rome, Italy; 4 National Agency for the Evaluation of Universities and Research (ANVUR), Rome, Italy; 5 St. Peter Fatebenefratelli Hospital, Rome, Italy

**Keywords:** Mandibuloacral dysplasia type A (MADA), Prelamin A forms, SUN2, Heterochromatin defects, Statins, Trichostatin A

## Abstract

Mandibuloacral dysplasia type A (MADA) is a rare laminopathy characterized by growth retardation, craniofacial anomalies, bone resorption at specific sites including clavicles, phalanges and mandibula, mottled cutaneous pigmentation, skin rigidity, partial lipodystrophy, and insulin resistance. The disorder is caused by recessive mutations of the *LMNA* gene encoding for A-type lamins. The molecular feature of MADA consists in the accumulation of the unprocessed lamin A precursor, which is detected at the nuclear rim and in intranuclear aggregates. Here, we report the characterization of prelamin A post-translational modifications in MADA cells that induce alterations in the chromatin arrangement and dislocation of nuclear envelope-associated proteins involved in correct nucleo-cytoskeleton relationships. We show that protein post-translational modifications change depending on the passage number, suggesting the onset of a feedback mechanism. Moreover, we show that treatment of MADA cells with the farnesyltransferase inhibitors is effective in the recovery of the chromatin phenotype, altered in MADA, provided that the cells are at low passage number, while at high passage number, the treatment results ineffective. Moreover, the distribution of the lamin A interaction partner SUN2, a constituent of the nuclear envelope, is altered by MADA mutations, as argued by the formation of a highly disorganized lattice. Treatment with statins partially rescues proper SUN2 organization, indicating that its alteration is caused by farnesylated prelamin A accumulation. Given the major role of SUN1 and SUN2 in the nucleo-cytoskeleton interactions and in regulation of nuclear positioning in differentiating cells, we hypothesise that mechanisms regulating nuclear membrane–centrosome interplay and nuclear movement may be affected in MADA fibroblasts.

**Electronic supplementary material:**

The online version of this article (doi:10.1007/s00418-012-0977-5) contains supplementary material, which is available to authorized users.

## Introduction

Mandibuloacral dysplasia type A [MADA; Online Mendelian Inheritance in Man (OMIM) no. 248370] is a rare and complex laminopathy characterized by postnatal growth retardation, craniofacial anomalies, bone resorption at specific sites including clavicles, phalanges and mandibula, mottled cutaneous pigmentation, partial lipodystrophy (type A pattern), and insulin resistance (Novelli et al. 2002). The disorder is caused by recessive mutations of the *LMNA* gene on chromosome 1q21.2 encoding for A-type lamins, including lamin A, lamin C, lamin A delta 10, and lamin C2 obtained by alternative RNA splicing (Maraldi et al. 2011).

Lamin A forms polymers at the nuclear lamina with lamin C. While lamin C is produced as mature protein, lamin A is translated as a precursor protein, which undergoes four steps of post-translational modifications, including farnesylation, double endoprotease cleavage and carboxymethylation (Maraldi et al. 2011). These modifications occur at the C-terminal Caa*X* motif, a sequence shared by farnesylated proteins, in which C is cysteine, the target of protein farnesyl transferase which catalyses prelamin A farnesylation. In human prelamin A, the aa*X* sequence consists of a serine, an isoleucine and a methionine (SIM residues) and the methionine directs the addition of the 15 Carbon farnesyl residues to cysteine. Following farnesylation, the aaX tripeptide is cleaved by ZMPSTE24 (zinc-dependent metalloproteinase Ste24 homolog) or RCE1 (Ras converting enzyme 1) and the C-terminal cysteine was carboxymethylated by the carboxymethyltransferase Icmt. The second ZMPSTE24-mediated cleavage of 15 amino acids at the C-terminus of prelamin A leads to removal of the farnesyl residue and yields mature lamin A (Dominici et al. 2009). Prelamin A processing is altered in laminopathies featuring premature aging and/or lipodystrophy, including Hutchinson–Gilford progeria (HGPS), Werner syndrome, restrictive dermopathy, familial partial lipodystrophy (FPLD2) and MADA, as well as in mandibuloacral dysplasia associated with mutations of the ZMPSTE24 endoprotease gene (MADB) (Maraldi and Lattanzi 2007). Prelamin A was postulated to be toxic for the cells and its toxicity has been attributed to the farnesylated residue. In agreement with this hypothesis, drugs impairing protein farnesylation have been shown to ameliorate the nuclear morphological abnormalities in laminopathic cells accumulating prelamin A and the whole phenotype in Zmpste24 null mice (Davies et al. 2011). It has been shown that reducing mutated prelamin A levels in progeria cells by splicing correction restores heterochromatin markers (Scaffidi and Misteli 2005). Moreover, we previously showed that in progeria cells accumulating farnesylated prelamin A, chromatin organization and function can be recovered by treating with mevinolin (an inhibitor of the hydroxymethyl-glutaryl-synthase eventually impairing prelamin A farnesylation) in combination with the inhibitor of histone deacetylases trichostatin A (TSA) (Columbaro et al. 2005). In the present study, we determine the post-translational modifications harbored by prelamin A in MADA cells and the effects of the treatment with mevinolin alone and in combination with TSA on heterochromatin. Here, we show that low passage fibroblasts from MADA patients accumulate farnesylated prelamin A. However, at high passage number, full-length prelamin A, possibly in its farnesylated and non-farnesylated forms, is detected in cells. The examined drug treatments appear to be effective in reducing heterochromatin defects in low passage cells only, possibly depending on the relative amount of prelamin A forms which are accumulated. Recovery of the cellular phenotype is demonstrated by changes in altered nuclear markers, such as trimethylated histone H3K9.

Moreover, the highly disorganized lattice formed by the nuclear envelope protein SUN2 in MADA nuclei (Mattioli et al. 2011) is rescued by treatment with mevinolin, indicating that the altered pattern of SUN2 distribution in the nuclear envelope of MADA fibroblasts is caused by farnesylated prelamin A accumulation.

## Materials and methods

### Cell cultures

MADA skin fibroblasts were obtained from patients carrying the homozygous R527H *LMNA* mutation that has been previously reported (Filesi et al. 2005; Lombardi et al. 2007). Control skin fibroblast cultures were obtained from skin biopsies of healthy patients (mean age 24) undergoing orthopedic surgery, following a written consent. The protocol had been approved by the local ethical committees. Cell cultures had been established and maintained in Dulbecco’s modified Eagle’s medium supplemented with 10 % fetal calf serum (FCS) and antibiotics. The experiments were performed at passages 8 or 14 (low passage samples) or 25 (high passage samples).

### Drug treatments

Impairment of farnesylation and further processing of prelamin A was achieved by the use of mevinolin, an inhibitor of the hydroxymethyl-glutaryl-synthase, applied at 25 μM concentration for 18 h. When specified, following mevinolin administration, cells were treated with 2.5 μM trichostatin A (TSA), an inhibitor of histone deacetylases, for additional 24 h.

### Antibodies

Antibodies employed for Western blot analysis or immunofluorescence labelling were: anti-lamin A/C, goat polyclonal (Santa Cruz, SC-6215, used at 1:1,000 dilution for the Western blot analysis); anti-prelamin A, goat polyclonal (Santa Cruz, SC-6214, lot. J3105 used at 1:100 dilution for the immunofluorescence analysis and at 1:700 dilution for the Western blot analysis); anti-prelamin A, rabbit polyclonal, raised against the last 18 aminoacids of the prelamin A sequence including the SIM sequence (antibody 1188-1, Diatheva); anti-prelamin A, rabbit polyclonal, raised against the last 15 aminoacids of the prelamin A sequence including the farnesylated cysteine residue but not the SIM sequence (antibody 1188-2, Diatheva); anti-trimethyl-H3K9, rabbit polyclonal (Upstate); anti-emerin, mouse monoclonal (Novocastra); anti-SUN1 or SUN2 rabbit polyclonal (Atlas); anti-actin, goat polyclonal (Santa Cruz).

### Western blot analysis

Western blot analysis was done as follows: cells were lysed in Ripa buffer containing 1 % Nonidet P-40, 0.25 % sodium deoxycholate, 0.1 % SDS, 10 mM Tris–HCl pH 7.4, 150 mM NaCl, 1 mM EDTA, 1 mM PMFS, 1 μM aprotinin, leupeptin, and pepstatin. Cell lysates were diluted in Laemmli buffer, subjected to SDS-PAGE (6–20 %) and transferred to nitrocellulose membrane. Membranes were saturated with 4 % BSA and incubated with primary antibodies for 1 h at room temperature at 1:100 dilution. Secondary antibodies were used at 1:10,000 dilution for 30 min. Immunoblotted bands were revealed by Amersham ECL detection system.

### Immunofluorescence

Human fibroblasts grown on coverslips were fixed in 4 % paraformaldehyde at 4 °C for 10 min and permeabilized with 0.15 % Triton X-100 for 5 min at room temperature. Alternatively, cells were fixed in absolute methanol at −20 °C. Samples were incubated with PBS containing 4 % BSA to saturate non-specific binding, incubated with primary antibodies overnight at 4 °C, and with secondary antibodies for 1 h at room temperature. Anti-prelamin A Sc-6214, anti-prelamin A 1188-1, anti-lamin A/C, anti-SUN1, anti-SUN2, and anti-trimethyl H3K9 were used at 1:100 dilution. Anti-prelamin A 1188-2 was applied at 1:10 dilution. Slides were mounted with DABCO (Sigma-Aldrich) anti-fading reagent in glycerol and observed with a Nikon E 600 fluorescence microscope equipped with a digital camera.

## Results

### Carboxymethylated prelamin A is accumulated in low passage MADA fibroblasts

**Fig. 1:**
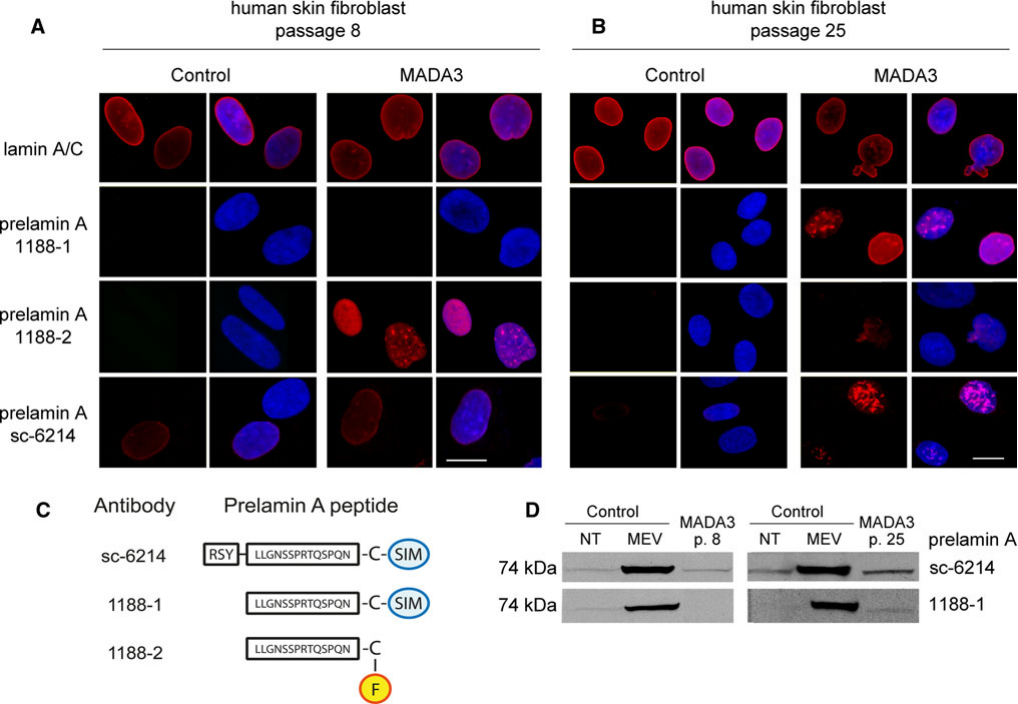
Prelamin A accumulation in low or high passage MADA cells. Immunofluorescence analysis of control or MADA fibroblasts at passage 8 (**a**) and at passage 25 (**b**) Lamin A/C was labelled by polyclonal anti-lamin A/C. Prelamin A was labelled by 1188-1, 1188-2 or Sc-6214 antibody, as indicated. *Bar* 10 μm. **c** Schematic representation of prelamin A epitopes that have been employed to raise the antibodies used in the present study. Specific sequences are indicated; F stands for farnesyl group. **d** Western blot analysis of control, mevinolin-treated or MADA fibroblasts. Prelamin A was labelled by Sc-6214 or 1188-1 antibody. Representative pictures of at least three experiments are shown

To discriminate the prelamin A form accumulated in MADA cells, we used two antibodies capable of detecting different prelamin A modifications (Fig. 1). Antibody 1188-1 binds full-length prelamin A either in its unprocessed non-farnesylated form, or harboring the farnesyl residue (first processing step) (Dominici et al. 2009). Antibody 1188-2 selectively detects farnesylated prelamin A devoid of the C-terminal SIM sequence (Dominici et al. 2009; Mattioli et al. 2011). Interestingly, immunofluorescence analysis performed on low passage cells revealed that antibody 1188-1 failed to label MADA cells, while antibody 1188-2 stained both the nuclear rim and inner nuclear invaginations (Fig. 1a). Antibody 1188-2 also labelled the nucleoplasm. As expected, control skin fibroblasts were negative for prelamin A staining using either antibody. At this stage, the Sc-6214 antibody, binding to any form of prelamin A, barely detected the precursor at the nuclear rim of MADA fibroblasts, and it faintly stained control nuclei. Thus, we suggest that farnesylated and partially processed (first step) prelamin A is accumulated in MADA cells at the first cell culture passages, while other prelamin A forms are not detectable at this stage.

### Full-length prelamin A is accumulated in high passage MADA fibroblasts

**Fig. 2:**
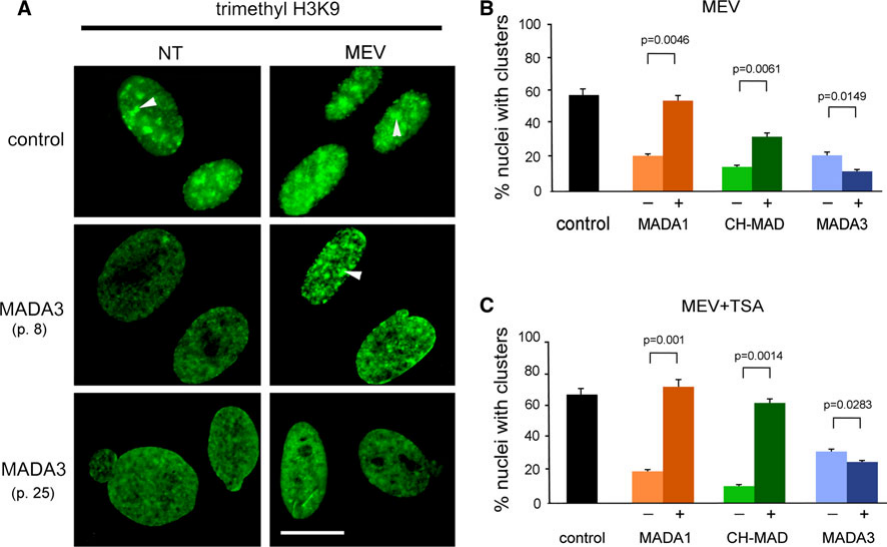
Treatment of MADA cell lines with mevinolin affects tri-methylated H3K9 labelling pattern. **a** Control or MADA3 cells at passage 8 or passage 25 were cultured in the absence or presence of 20 μM mevinolin for 18 h. Tri-methylated H3K9 (tri-H3K9) labelling pattern is shown. Clusters of tri-H3K9 are indicated by arrowheads. *Bar* 10 μm. *NT* non-treated, *MEV* fibroblasts treated with mevinolin. **b**, **c** Statistical analysis. The *graphs* show the percentage of nuclei showing clustered trimethylated-H3K9 in control and MADA cells. MADA cells were left untreated or treated (*black* and *colored bars*, respectively) with increasing concentrations of mevinolin (25 μΜ) either alone (**b**) or followed by TSA (2.5 μM) (**c**). The study includes one control and three MADA cell lines: MADA1 (homozygous R527H *LMNA* mutation, age 18 years), CH-MADA (heterozygote compound R527H/V440M *LMNA* mutation, age 27 years), MADA3 (homozygous R527H *LMNA* mutation, age 50 years). Values are means of two independent experiments ± standard deviation; for each experiment a total of 100 nuclei were taken into consideration; *p* values indicate statistical significance of the difference between treated and non-treated samples

Immunofluorescence analysis of MADA fibroblasts at passage 25 showed a markedly reduced labelling by 1188-2 antibody (Fig. 1b). The nuclear envelope and the nucleoplasm were faintly labelled by this antibody and no staining at nuclear invaginations could be observed. On the contrary, distinct staining with 1188-1 antibody, which binds full-length prelamin A (Fig. 2c), was observed both at the nuclear rim and at intranuclear invaginations. These prelamin A aggregates likely represent nuclear lamina invaginations, as suggested by previous studies (Filesi et al. 2005; Lattanzi et al. 2007; Mattioli et al. 2008).

Western blot analysis of both low and high passage cells revealed that a prelamin A band was clearly detectable by 1188-1 or Sc-6214 antibody in MADA fibroblast at passage 25 (Fig. 2d). These data suggest that full-length prelamin A is accumulated in high passage MADA fibroblasts, while carboxymethylated prelamin A level appears to be reduced at this stage.

### Drug treatments rescue tri-methyl H3K9 pattern in low passage cells

Cells from progeroid laminopathies are known to present chromatin defects, consisting of peripheral heterochromatin loss and disorganization of heterochromatin sites labelled by tri-methylated histone 3 on lysine 9 (tri-H3K9) and HP1 (Columbaro et al. 2005; Filesi et al. 2005; Larson et al. 2012). Certain drug treatments including farnesyltransferase inhibitor administration or addition of mevinolin in combination or not with TSA to cell cultures have been previously shown to rescue the chromatin phenotype in progeria cells (Columbaro et al. 2005; Galiova et al. 2008). In order to define the efficacy of these pharmaceutical tools in MADA cells, we considered the labelling pattern of tri-H3K9 as a parameter, and monitored its staining before and after drug treatment. Administration of mevinolin rescued the labelling pattern of tri-H3K9 in low passage MADA fibroblasts, while treatment was not effective in high passage cells (Fig. 2a, middle and bottom row, respectively). In fact, in mevinolin-treated MADA cells at passage 8, the diffuse staining characteristic of untreated MADA nuclei at any passage number was changed to intranuclear clusters, comparable to the pattern observed in control nuclei. However, the diffuse tri-H3K9 staining of MADA fibroblasts at passage 25 was not improved by the drug (Fig. 2a, bottom row). The different responsiveness of the three MADA cell cultures to drug treatments is shown in Fig. 2b and c. As shown by the statistical analysis, the age of patient at biopsy, but not the mutation, influenced drug efficacy. TSA administration, following mevinolin treatment, induced an even more pronounced improvement, indicating a positive synergistic effect on the rescue of chromatin organisation (Fig. 2c).

### Drug treatment rescues SUN2 organization in MADA cells

**Fig. 3:**
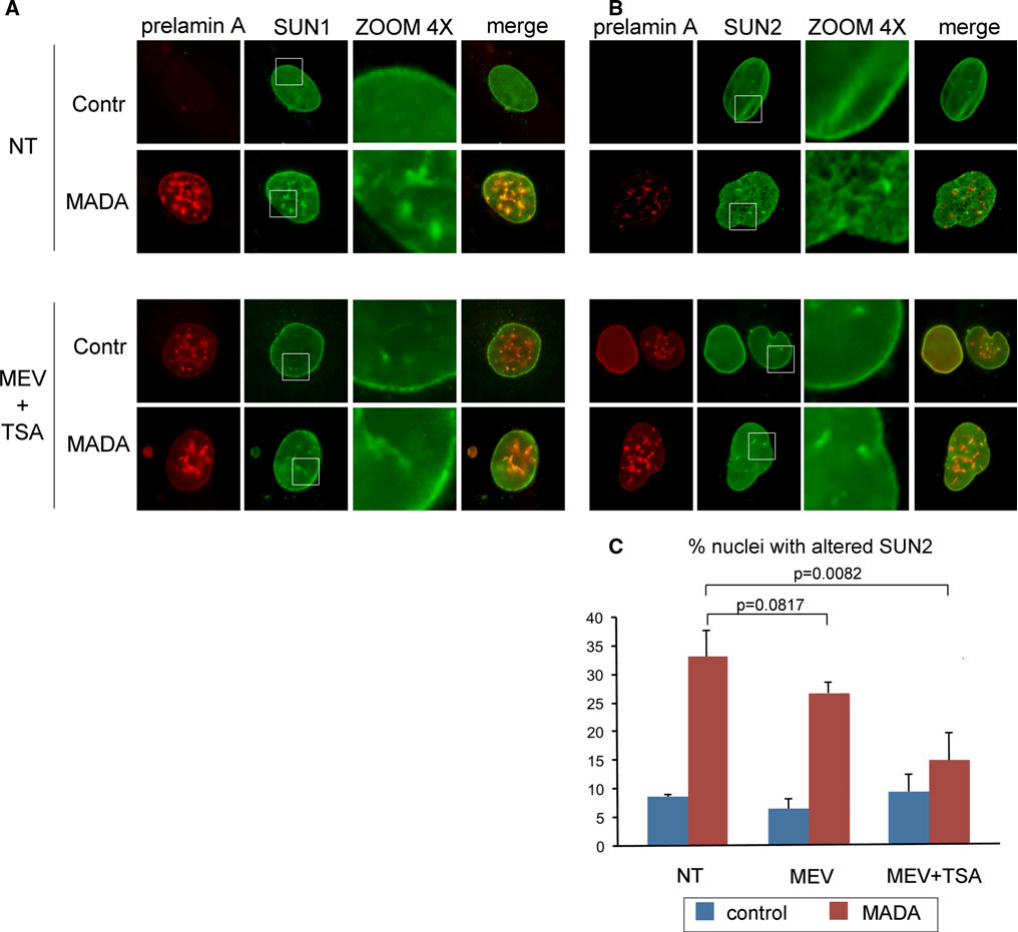
SUN2 organization at the nuclear envelope is rescued by drug treatment. Control and patient fibroblasts were cultured in the presence or absence of mevinolin (25 μM for 18 h). Cells were fixed in ice cold methanol and immunofluorescence assay was performed using goat anti-prelamin A and rabbit SUN1 or SUN2 antibodies. Cell nuclei were counterstained with DAPI. Panels **a** and **b** show SUN1 and SUN2 staining, respectively, in control and treated conditions as indicated (*NT* non-treated, *MEV* *+* *TSA* treated with mevinolin and trichostatin A). **c** The *graph* represents the percentage of nuclei showing SUN2 in a honeycomb-like pattern in both control and MADA fibroblasts, before and after treatment with mevinolin (25 μM for 18 h) and mevinolin (25 μM for 18 h) followed by trichostatin A (2.5 μM for 24 h) (NT, MEV and MEV + TSA, respectively). Nuclei were considered positive to SUN2 alteration when displaying a honeycomb pattern on the nuclear surface. Experiments were performed 3 times and a minimum of 200 nuclei was counted per sample. Values are means of three independent experiments ± standard deviation; *p* values indicate the statistical significance of the difference of treated MADA samples compared with the untreated one

SUN1 and SUN2 are nuclear membrane proteins known to interact with prelamin A (Crisp et al. 2006; Mattioli et al. 2011). Immunofluorescence assays in MADA fibroblasts revealed altered distribution of both proteins. In fact, SUN1 was properly localized at the nuclear envelope, but it was also found at intranuclear invaginations. Prelamin A aggregates were found to colocalize with SUN1-labeled nuclear invaginations. Further, SUN2 was mislocalized forming a honey-comb-like lattice in the nuclear membrane (Fig. 3a, b). SUN1 and SUN2 disorganization persisted in high passage MADA fibroblasts (not shown).

In line with the observation of a rescue in chromatin organisation by drug administration, we applied mevinolin and mevinolin/trichostatin A treatment to MADA fibroblast and obtained an improvement of the labelling pattern of SUN2 in MADA nuclei that lost the honeycomb pattern in favor of a more homogenous distribution despite a persistent co-localization with prelamin A aggregates (Fig. 3b, c). Differently, SUN1 localization in intranuclear aggregates resulted unaffected (Fig. 3a).

Comparable results in SUN2 pattern recovery were obtained in MADA cells independent from the number of passages in culture, suggesting that, differently from what observed for the tri-methylated H3K9, drug treatment was efficient for phenotype rescue also at high passage number. However, complete rescue was not achievable as indicated by the persistence of co-localization with prelamin A aggregates for both SUN2 and SUN1 in control, mevinolin and mevinolin plus trichostatin A treatment conditions. Taken altogether, these data suggest that SUN2 is mainly affected by accumulation of farnesylated prelamin A and its reduction by drug treatment mediates partial recovery of phenotype at any stage of disease progression.

## Discussion

The reported study shows that MADA fibroblasts accumulate different forms of prelamin A depending on the passage number. Farnesylated prelamin A is accumulated in low passage cells, while full-length prelamin A, either farnesylated or non-farnesylated, is detectable in high passage fibroblasts. We further show that drugs interfering with prelamin A processing can rescue the proper distribution of the heterochromatin marker tri-H3K9 and the organization of the nuclear envelope protein SUN2 in MADA fibroblasts.

Data so far reported in laminopathic cells strongly suggest that trimethylation of lysine 9 on histone 3 is a hallmark of chromatin defects related to lamin A mutations. In fact, tri-H3K9 aberrant organisation, manifested by loss of clustered distribution and reduced labelling intensity, has been reported in HGPS, RD and MADA cells (Columbaro et al. 2005, 2010; Filesi et al. 2005; Shumaker et al. 2006). Moreover, it has been shown that a direct correlation exists between levels of farnesylated prelamin A in the nuclear lamina and degree of H3K9 methylation (Columbaro et al. 2005, 2010; Lattanzi et al. 2007; Mattioli et al. 2008; Shumaker et al. 2006). Most importantly, it has been demonstrated that reduction of prelamin A levels or impairment of its farnesylation in laminopathic cells improves tri-H3K9 labelling pattern within the nucleus, thus supporting the intriguing hypothesis that some enzymatic activity regulating histone 3 methylation may be affected by prelamin A accumulation (Columbaro et al. 2005).

Here, we demonstrate that rescue of tri-H3K9 methylation can be achieved in MADA fibroblasts at low passage number, through a treatment aimed at reducing lamin A precursor farnesylation. However, the onset of an unknown mechanism, leading to accumulation of full-length prelamin A (either farnesylated or not) in high passage MADA fibroblasts, makes ineffective the treatment of cells with mevinolin. The most likely explanation for these results is that the cell requires a constant rate of farnesylated to non-farnesylated prelamin A, which is unbalanced towards farnesylated prelamin A in low passage MADA fibroblasts, while it is unbalanced towards non-farnesylated prelamin A in high passage cultures. Thus, knowledge of the mechanism, which affects prelamin A processing in MADA, is needed in order to efficiently re-establish the physiological condition. Moreover, other nuclear envelope markers here used to monitor the effects of *LMNA* mutations on nuclear organization are two trans-membrane proteins, namely SUN1 and SUN2, which are known binding partners of prelamin A (Haque et al. 2006, 2010; Crisp et al. 2006; Mattioli et al. 2011). SUN1-increased expression has been correlated with accumulation of farnesylated prelamin A in progeria cells (Haque et al. 2010). Here, we show that MADA cells, reported to accumulate prelamin A in intranuclear aggregates (Capanni et al. 2005, Filesi et al. 2005), display a partial recruitment of SUN1 in these aggregates, overlapping with chromatin decondensation areas. Interestingly, SUN2, which does not appear to be altered in HGPS, is mislocalized in MADA. Importantly, SUN2 mislocalization in honeycomb structures is partially recovered after drug treatment, suggesting that it is mostly dependent on the accumulation of farnesylated prelamin A. To be noted, differently from what observed for the tri-H3K9, defarnesylating treatment is shown to be effective on SUN2 rescue in MADA fibroblasts at any passage number, thus implying an even stronger relation between loss of farnesylated precursor, in both its partial or non-processed form, and recovery of proper distribution. Importantly, similarly to SUN2, an altered distribution of the nuclear envelope protein emerin has been previously reported in MADA (Filesi et al. 2005). Thus, we suggest that prelamin A accumulation in MADA interferes with both heterochromatin organization and a variety of inter-molecular interactions required for proper nuclear envelope assembly.

It is known that specific tissues are prevailingly targeted in MADA: adipose tissue, skin, and certain bone segments. As in other systemic laminopathies, the accumulation of abnormal amounts of prelamin A, besides causing alteration in the heterochromatin pattern, can specifically interfere with the availability of tissue-specific transcription factors. This has been previously demonstrated in MADA fibroblasts, in which prelamin A aggregates resulted in a reduced availability of free SREBP1, thus affecting the expression of genes involved in adipogenic differentiation (Capanni et al. 2005).

The study here reported suggests an additional pathogenic mechanism implicating a role for the different modification status of NE proteins; however, the pathogenetic significance of SUNs mislocalization in relation to the nature of prelamin A form accumulated in MADA is not obvious. It has been suggested that alterations in lamin A/C might compromise interactions between the nucleus and cytoskeleton involved in nuclear positioning (Mejat et al. 2009; Mattioli et al. 2011). In fact, *LMNA* mutations have been reported to affect the linkage of the nucleus to the cytoskeleton (Schneider et al. 2010; Hale et al. 2008; Luxton et al. 2011). In a recent study, by examining nuclear movement during centrosome orientation in migrating fibroblasts, which requires A-type lamins, it has been determined that *LMNA* mutations causing muscle diseases, block actin-dependent nuclear movement, whereas most that affect adipose tissue, such as MADA, inhibit microtubule-dependent centrosome positioning (Folker et al. 2011). Besides adipose tissue, MADA patients present a mottled cutaneous pigmentation and a sclerotic skin and osteolysis. The biological mechanism causing osteolysis in some bone segments of MADA patients is not completely understood. However, some aspects of the osteolytic process have been shown to be altered in either animal or cellular models of MADA (Avnet et al. 2011; Duque et al. 2011). We have recently provided evidence that MADA osteoblast-derived factors affect osteoclast differentiation and activity, suggesting that the altered bone turnover in MADA is due to up-regulation of osteoblast-derived stimulatory cytokines, including TGF beta 2, that affect osteoclast activity (Avnet et al. 2011). We previously reported that accumulation of prelamin A induced by drugs interfering with its processing results into increased osteoclast differentiation from human peripheral blood monocytes (Zini et al. 2008). Here, we propose that mislocalization of SUN1 and SUN2 occurring in MADA cells contributes to dysregulation of the mechanism of cell fusion, which is required for osteoclast formation. Further, a general mechanism of nuclear positioning implicated in bone, adipocyte and muscle differentiation and depending on SUN proteins and the LINC complex (Folker et al. 2011; Mattioli et al. 2011; Starr 2009) may play a role in MADA pathogenesis.

The results here reported also suggest that, whichever pathogenic mechanism could be activated through farnesylated prelamin A accumulation, mevinolin, and mostly mevinolin plus TSA (Columbaro et al. 2005), are effective in the recovery of both chromatin arrangement and of proper SUN2 organization.

The effects of drug treatments reported in this study give new important insights. First, molecules involved in MADA pathogenesis are not only those regulating chromatin organization but contingently also the integral membrane proteins SUN1 and SUN2 and likely other factors implicated in nuclear positioning and nucleo-cytoskeleton interplay, a relevant aspect in adipose tissue, skin, and bone development, which are target tissues in MADA patients. Second, improvement of the cellular phenotype by statin or statin/TSA treatment is better reached in low passage cells, when only the farnesylated form of prelamin A is accumulated. The latter finding further implies that simultaneous accumulation of different prelamin A processing intermediates might worsen the cellular phenotype and result into a less tractable pathology. Further, these data, and the evidence that drug-mediated improvement of the nuclear phenotype is also influenced by patient age at biopsy, may help explaining the efficacy of current clinical trials with statins in MADA and the less satisfactory response observed in older patients.

Moreover, this study shows how different protein partners are differently affected by drug treatments, supporting a picture of an intricate network of finely regulated interactions involved in pathogenetic mechanisms. In this context, the global restoration of this variety of functional interactions in which prelamin A plays a central role, appears even more complicated, raising the need of more than a univocal strategy (Richards et al. 2011) for effective treatment.

## Electronic supplementary material

Below is the link to the electronic supplementary material.

Supplementary material 1 (DOC 127 kb)
