# Supplementary material for: Altered chromatin organization and SUN2 localization in mandibuloacral dysplasia are rescued by drug treatment
Source: Histochem Cell Biol. 2012 Jun 17;138(4):643–51. doi: 10.1007/s00418-012-0977-5 (PMC3432780; doi:10.1007/s00418-012-0977-5)

**Supplementary material**

**Fig. S1. Count of nuclei positive for clustered tri-methylated H3K9.**

The tables indicate the counts of trimethylated H3K9 positive nuclei in MADA samples non treated and treated with mevinolin (S1 A) or mevinolin plus TSA (S1 B) compared with control cells. The figure indicates the positive nuclei over 200 in total, obtained with two separated experiments. Table A and B report the numbers of counted cells corresponding to the graphs shown in Fig. 2A and 2B, respectively.

**FIG. S2. Count of nuclei with altered SUN2 in MADA and control cells in the absence/presence of drug treatment.**

Nuclei of control and MADA cells non treated or treated with mevinolin or mevinolin plus TSA were analyzed for proper distribution of SUN2 at the nuclear envelope. Numbers correspond to the counts of nuclei showing SUN2 alteration over the total nuclei considered from three different experiments. Data correspond to the graphic representation reported in Fig. 3C.

**Fig. S1**

**A**


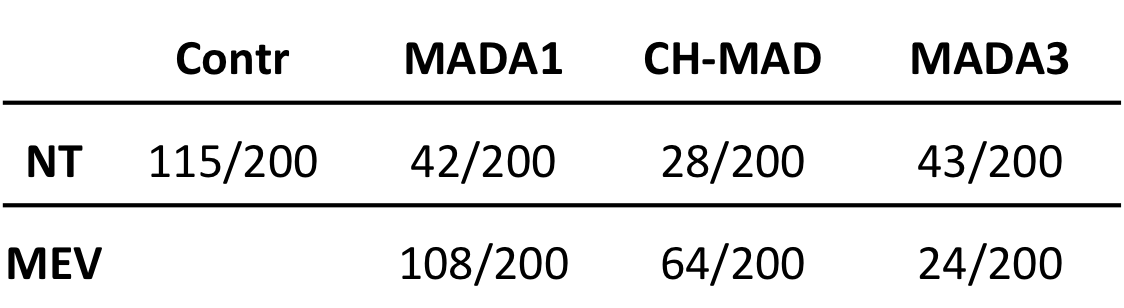


**B**


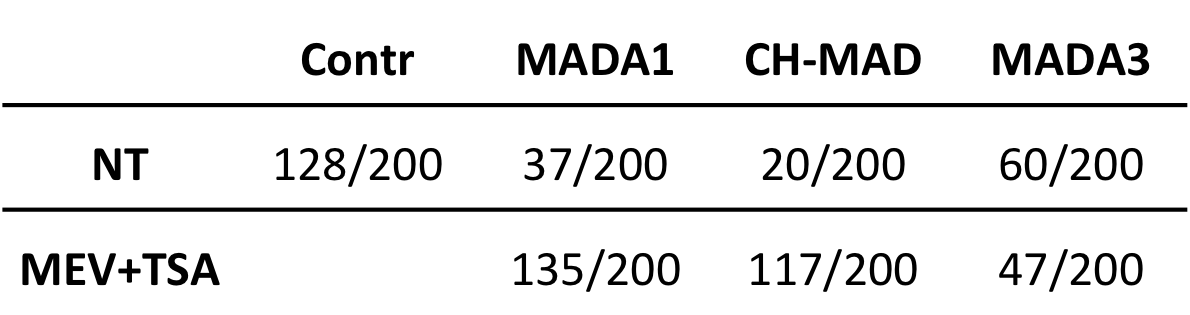


**Fig. S2**


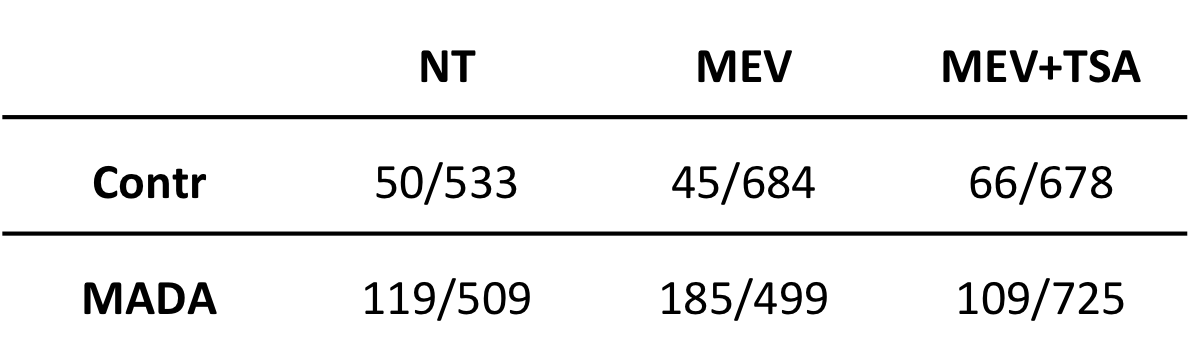

Supplement: Supplementary file 1 — Supplementary material 1 (DOC 127 kb) [file 418_2012_977_MOESM1_ESM.doc]
